# Development and Evaluation of a Software Designed by a Nursing and Technology Team to Assess the Health Status of Adults over 65 Years of Age

**DOI:** 10.17533/udea.iee.v42n2e07

**Published:** 2024-07-04

**Authors:** Víctor Pérez Cantó, Víctor M. González Chorda, Francisco Miguel Escandell Rico, Manuel Platero Platero Horcajadas, Francisco Javier Ferrández Pastor, Ana Castillo López, María Jesús Valero Chillerón, Loreto Maciá Soler

**Affiliations:** 1 Nurse, Ph.D. Professor. Hospital VITHAS Perpetuo Socorro, Alicante; Spain. Email: victor.pc@ua.es Universidad de Alicante Hospital VITHAS Perpetuo Socorro Alicante Spain victor.pc@ua.es; 2 Nurse, Ph.D. Professor, Universitat Jaume I, Castelló de la Plana; Spain. Email: vchorda@uji.es Universitat Jaume I Universitat Jaume I Castelló de la Plana Spain vchorda@uji.es; 3 Nurse, Ph.D. Professor. Email: francisco.escandell@ua.es Universidad de Alicante Spain francisco.escandell@ua.es; 4 Computer Engineer, PhD candidate. Email: manuel.platero@ua.es Universidad de Alicante Spain manuel.platero@ua.es; 5 Industrial Engineer, Ph.D. Professor. E-mail Fjferran@ua.es Universidad de Alicante Spain Fjferran@ua.es; 6 Nurse, Masters. Professor, Universidad Cardenal Herrera, Elche; Spain. E-mail: anacl90.ac@gmail.com Universidad Cardenal Herrera Universidad Cardenal Herrera Elche Spain anacl90.ac@gmail.com; 7 Nurse, PhD. Professor, Universitat Jaume I, Castellón; Spain. Email: chillero@uji.es Universitat Jaume I Universitat Jaume I Castellón Spain chillero@uji.es; 8 Nurse PhD. Professor. Email: loreto.macia@ua.es. Corresponding author. Universidad de Alicante Spain loreto.macia@ua.es; 9 Universidad de Alicante; Spain Universidad de Alicante Universidad de Alicante Spain

**Keywords:** aged, frailty, software, information systems, health management, needs assessment., adulto mayor, fragilidad, programas informáticos, sistemas de información, gestión en salud, evaluación de necesidades., idoso, fragilidade, software, sistemas de informação, gestão em saúde avaliação das necessidades.

## Abstract

**Objective.:**

This work sought to develop the *Actuasalud* platform as a useful tool for nursing that permits assessing health, in term of frailty, in population over 65 years of age.

**Methods.:**

For the design and development of *Actuasalud*, two working groups were formed: one from nursing with different profiles, to identify the scientific content and a computer science group responsible for the software programming and development. Both teams adapted the scientific content to the technology so that the tool would allow for population screening with detection of health problems and frailty states.

**Results.:**

The software was developed in three large blocks that include all the dimensions of frailty: a) sociodemographic variables, b) comorbidities, and c) assessment tools of autonomy-related needs that evaluate the dimensions of frailty. At the end of the evaluation, a detailed report is displayed through bar diagram with the diagnosis of each of the dimensions assessed. The assessment in the participating elderly showed that 44.7% (*n* = 38) of the population was considered not frail, and 55.3%; *(n* = 47) as frail. Regarding associated pathologies, high blood pressure (67.1%; *n* = 57), osteoarthritis and/or arthritis (55.3%; *n* = 47), diabetes (48.2%; *n* = 41) and falls during the last year (35.3%; *n* = 30) were highlighted.

**Conclusion.:**

*Actuasalud* is an application that allows nursing professionals to evaluate frailty and issue a quick diagnosis with ordered sequence, which helps to provide individualized care to elderly individuals according to the problems detected during the evaluation.

## Resumen

**Objetivo.:**

Desarrollar la plataforma *Actuasalud* como una herramienta útil para enfermería que permita evaluar la salud, en términos de fragilidad, en población mayor de 65 años.

**Métodos.:**

Para el diseño y desarrollo de *Actuasalud*, se constituyeron dos grupos de trabajo: uno de enfermería con diferentes perfiles para identificar el contenido científico y uno informático que se responsabilizó de la programación y desarrollo del software. Ambos equipos adaptaron el contenido científico a la tecnología de manera que la herramienta permitiese hacer un cribado poblacional con detección de problemas de salud y estados de fragilidad.

**Resultados.:**

Se desarrolló el software en tres grandes bloques que incluyen todas las dimensiones de fragilidad: a) variables sociodemográficas, b) comorbilidades y c) herramientas de evaluación de necesidades relacionadas con la autonomía que evalúan las dimensiones de fragilidad. Al finalizar la evaluación, se visualiza un informe detallado mediante diagrama de barras con el diagnóstico de cada una de las dimensiones evaluadas. La evaluación en los mayores participantes mostró que el 44.7% (*n* = 38) de la población se consideró como no frágil, y un 55.3%; *(n* = 47) como frágiles. En cuanto a las patologías asociadas, destacaron hipertensión arterial (67,1 %; *n* = 57), artrosis y/o artritis (55.3%; *n* = 47), diabetes (48.2 %; *n* = 41) y caídas en el último año (35,3 %; *n* = 30).

**Conclusión.:**

*Actuasalud* es una aplicación que permite a los profesionales de enfermería evaluar fragilidad y emitir un diagnóstico de forma ágil con secuencia ordenada que ayuda a brindar cuidados individualizados a personas mayores de acuerdo los problemas detectados en la evaluación.

## Resumo

**Objetivo.:**

Desenvolver a plataforma Actuasalud como uma ferramenta útil para a enfermagem que permite avaliar a saúde, em termos de fragilidade, numa população com mais de 65 anos.

**Métodos.:**

Para a concepção e desenvolvimento do Actuasalud foram formados dois grupos de trabalho: um grupo de enfermagem com perfis diferentes, para identificar o conteúdo científico, e um grupo de informática que foi responsável pela programação e desenvolvimento do software. Ambas as equipas adaptaram o conteúdo científico à tecnologia para que a ferramenta permitisse o rastreio da população para detectar problemas de saúde e estados de fragilidade.

**Resultados.:**

O software foi desenvolvido em três grandes blocos que incluem todas as dimensões da fragilidade: a) variáveis ​​sociodemográficas, b) comorbidades ec) instrumentos de avaliação de necessidades relacionadas à autonomia que avaliam as dimensões da fragilidade. Ao final da avaliação é apresentado um relatório detalhado através de um diagrama de barras com o diagnóstico de cada uma das dimensões avaliadas. A avaliação nos idosos mostrou que 44.7% (*n*=38) da população foi considerada não frágil e 55.3%; (*n*=47) como frágil. Quanto às patologias associadas, destacaram-se a hipertensão arterial (67.1%; *n*=57), a osteoartrite e/ou artrite (55.3%; *n*=47), a diabetes (48.2%; *n*=41) e as quedas no último ano (35.3%; *n*=30).

**Conclusão.:**

Actuasalud é um aplicativo que permite ao profissional de enfermagem avaliar a fragilidade e emitir um diagnóstico de forma ágil e com sequência ordenada que auxilia no atendimento individualizado ao idoso de acordo com os problemas detectados na avaliação.

## Introduction

Health systems face an epidemiological outbreak of people with an elderly profile, multimorbidity and complex care needs, which represent a clinical and social challenge. Factors, like demographic change, increased life expectancy, lifestyles or sustainability of health systems, make the importance of prevention and health promotion acquire significant dimensions to guarantee accessible, universal, and equitable health care.^1^In the 21^st^ century, digital resources provide the opportunity for communication to progressively reach all social levels, which means receiving immediate feedback for people. Nursing is not immune to this technological impact, but must seek a balance between the comprehensive care of individuals and the use of technologies that facilitate this task.

Information and communication technologies (ICTs) offer an opportunity for application in different areas from which the health sector is not exempt (e-health).^2^E-health programs contribute to enhance people-centered systems, improve public health capacity towards universal coverage, and enable quality care using technology and internet connectivity to improve health services.^2^ Digital technologies and, especially mobile and wireless technologies, are appropriate in the health field given their ease of use, dissemination, and acceptance. Specifically, the elderly, by increasing technological usability, are an ideal group to benefit from advances in the use of ICTs that can help prevent frailty.^3^ A survey conducted in 2022 by the Statistics National Institute (INE, for the term in Spanish) in Spain, related with technological usability, showed increased use of ICTs up to 85% in the elderly with 22% of the population over 75 years of age included in the survey data using the internet.^4^ This may be the reason why many commercial companies and researchers have taken advantage of ICTs to design applications aimed at promoting healthy habits that can also serve as social support for the elderly.^5^Most applications focusing on the elderly address health problems such as multiple medications, monitoring of vital signs or falls, among other aspects, but in a single-dimensional manner.^6^ Furthermore, the number of mobile applications that allow evaluating complex or frail situations and guaranteeing a comprehensive approach to nursing care is limited.^7^

To use the term frailty, we must go back to the 1980s when frailty was defined as a syndrome with a multiplicity of clinical-biological manifestations but no symptoms.^8^ Frailty is dynamic, potentially reversible, process, predictor and risk factor for disability and of serious adverse events where, once a state of disability is reached, it is no longer reversible and evolves towards dependency.^8^ Lack of consensus exists on the definition and screening methods for frailty, although by the late 1990s the World Health Organization defined as risk factors for frailty that of being over 80 years of age; having a disabling chronic illness; being institutionalized; suffering from comorbidities, disability, dependence, cognitive alterations, depression, frequent falls, loneliness, and risk of poverty.^1^

Given the lack of comprehensive approaches to understanding the health status of the elderly, the scarcity of tools to support decision making, and the need to increase prevention and health promotion actions in this group, it seems pertinent to develop comprehensive assessment tools, as is the case of the Actuasalud digital platform, a software created for the purpose of standardizing and improving the assessment of health conditions and frailty screening in the elderly population living in the community. It is a software aimed at nurses that allows assessing the state of health and frailty in people over 65 years of age from any mobile device.(^9^) The aim of this study was to introduce the development of the Actuasalud platform from its conception as research (R), its technological development (D), and innovation (I) as a useful tool for nursing that permits assessing health in population over 65 years of age and showing the results obtained from the assessment of the first sample of elderly evaluated.

## Methods

To create *Actuasalud*, a scientific team called Tecnosalud was organized at Universidad de Alicante in Spain, made up of two computer engineers and five nursing professionals with different profiles, two nurses with academic profile and > 5-year experience in public universities, two care nurses from the public primary health care system, and one nurse with private hospital management profile. To carry out the work, the scientific team was separated into two groups, a health group and a technology group. The coordination was performed by the senior academic nurse as head of the Tecnosalud team. Both groups established the conceptual bases for the construction of the *Actuasalud* platform: basic definition aspects of the application, development objectives, and contributions or results expected, as well as the objectives of each R+D+I phase. The research and development process was conducted between 2019 and 2020

The work of the health group consisted in conducting an exhaustive literature review to decide the content of the software with the best evidence on the environment in the assessment of the needs and syndromes that affect older adults prior to the development of the application. The study followed recommendations for the detection of alterations in human needs and frailty in primary care and in the community setting.^10^^,^^11^A selection of validated questionnaires and diagnostic tests was also made to assess health and provide a diagnosis related with frailty.^3^ Additionally, a selection of complementary tests was conducted to allow specific health problems to be diagnosed independently. This would permit implementing adequate strategies in care, health promotion, and disease prevention to each group of elderly individuals with similar problems. Upon constructing the *Actuasalud* contents, for its validation with individuals, the study had four healthcare nurses from a primary care center and who had not participated in the construction of the platform. During the first 85 evaluations, every time five health diagnoses were made, a consensus group compared the results of the evaluation with the observation by the nurse evaluator, which allowed correcting possible diagnostic errors.

**Figure 1:**
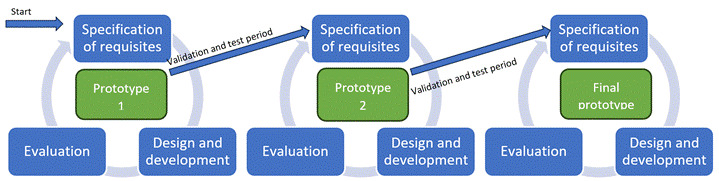
Phases of the development cycle of the *Actuasalud* software to assess health in the elderly

The software engineering used an agile application development method known as eXtreme Programming (XP).E.^12^The method followed was based on continuous feedback between nurses and the development team. Thus, dynamic and short development cycles were proposed. Through several meetings by both teams, the requirements and functionalities were agreed; these meetings were held periodically at the start of the development of each prototype. General phases in software development were followed to create the application’s prototypes;^12^hence, in each cycle, a complete process of defining requirements and needs, design and development, and evaluation was carried out (Figure 1).

*Design and development de la Actuasalud platform*

Upon establishing the system’s minimum requirements through meetings between the nursing and technology groups, it was decided on the construction of a digital platform aimed at nursing professionals to expedite the evaluation of non-institutionalized individuals > 65 years of age without cognitive impairment. The evaluation had to determine the health status of the individuals by obtaining information related with their autonomy and possible state of frailty, in this order (Frailty; cognitive, functional, and nutritional states; health-related quality of life; socio-family situation; affective state; mobility; vision; hearing; security; sleep; and pain);^9^ besides collecting socio-demographic data, comorbidities and pharmacological treatment. This permitted viewing a health diagnosis and formulating an individualized nursing care prescription. The order of the items and nursing assessment scales was determined; once the informed consent from each person is obtained, the evaluation begins with the socio-demographic data to continue with the comorbidities; prosthesis and drugs taken at the time of the evaluation to continue with the evaluation scales that the software should contain, as well as the scores that establish the differences among health states. 

The software was developed with two principal elements: (i) Element 1. Application programed with WEB technology using the IONIC cross-platform mobile programming framework; (ii) Element 2. Administration panel and an application programming interface (API) that permit managing the information shown in the application. This part was developed via PHP through the Symfony development framework, which is connected to a MYSQL database through the ORM Doctrine. The application’s research and development was carried out between March and September 2019 and innovation with knowledge transfer took place in November 2020. As of the moment of the knowledge transfer, the necessary steps to commercialize the platform were undertaken.^9^

*Tool validation*

To validate the tool, 85 individuals > 65 years of age without cognitive impairment and not institutionalized were evaluated. These subjects were contacted through nurses from primary care centers who decided to collaborate in the project. The assessment was performed by practicing clinical nurses who collaborated in the project through cooperation agreements with the university. To contrast the results of the assessments, six consensus groups organized made up of the nurse who had carried out the assessment, the person responsible for the software, an observer nurse, and the coordinator of the Tecnosalud team. The coordinator is an academic/research nurse with over five years of experience; the observer and evaluating nurses had over five years of clinical experience; and the person responsible for the software was a computer engineer with five years of experience. This group met once per week to validate that the result, of the assessments issued by *Actuasalud*, corresponded to the evaluator’s perception.^(13 )^ In case of disagreement between the score obtained in any dimension and the opinion of the evaluating nurse, its origin was analyzed to assess and even discard the evaluation with diagnostic purposes. 

*Statistical analysis*

A descriptive statistical analysis was conducted using the chi-square test for the prevalence of frailty and care needs, comparing socio-demographic and clinical variables in function of two levels of frailty (Not frail and Frail). Thereafter, multivariate logistic models were adjusted to estimate the magnitude of the associations, estimating the Odds Ratios (OR). Analyses were performed via SPSS v.26 and R v.4.0.0 packages. 

*Ethical considerations*

For the process of elaboration of *Actuasalud* and its validation, ethical and legal aspects have been followed according to the Declaration of Helsinki^14^ and Organic Legislation 3/2018, of December 5, on the Protection of Personal Data and guarantee of digital rights,^15^as well as Regulation (EU) 2016/679 by the European Parliament and by the Council of 27 April 2016, on the Protection of Individuals. Prior to each assessment, informed consent was requested from each participant.

Data stored in *Actuasalud* is encrypted through a private key certificate, which encrypts all open response evaluation data, such as the observation fields and prevents data from an assessment from being associated with a patient. The server and database where the data is stored has an access control system where all access attempts are recorded. Lastly, access to data from an assessment from the application is recorded and monitored through files that keep the registry of user actions in the application. Thus, it allows to verify which person has accessed certain data. For data protection, *Actuasalud* includes a module online management of informed consents to ensure rights of access, rectification, cancellation, and opposition. Furthermore, the system is designed to generate, without trace, the assignment of a random number that will be used as identification of the user’s assessment.

## Results

This study resulted in the development of the *Actuasalud* software, registered by Universidad de Alicante,^9^ transferred for its commercialization after having gone through all the technological maturity phases, using the Technology Readiness levels (TRL) scale from TRL1 (basic principles observed) to TRL9, real system tested in an operational environment (competitive manufacturing in the case of key enabling technologies). It can be assured that *Actuasalud* is able to evaluate autonomy, needs of daily life, and frailty states in population over 65 years of age without cognitive impair, with diagnostic report and data dump for subsequent analysis. 

### Software-related aspects

The *Actuasalud* platform comprises three large blocks: (i) Sociodemographic variables: age, sex, educational level, marital and socioeconomic status among others; (ii) Comorbidities; and (iii) Validated needs assessment scales that evaluate cognitive state, frailty, functional status, nutritional status, quality of life, socio-family situation, affective state, mobility, vision, hearing, safety (risk of falls and pressure ulcers), sleep, and pain.^3^ The forgoing are summarized in Table 1.

**Table 1:** Scales used to assess needs and number of items that compose them

Need assessed	Scale used	Nº of items
Frailty	Frail	18
Cognitive state	PFEIFFER’S test	11
Nutritional assessment	Nutrition Screening Initiative (NSI)	10
Health-related quality of life	EQ-5D questionnaire	6
Functional state. Autonomy for basic daily living activities	BARTHEL’S index	10
Functional state. Instrumental daily living activities	LAWTON-BRODY index	8
Social risk	GIJON scale	5
Affective state	YESAVAGE scale	15
Assessment of visual ability	VF-14 scale	14
Hearing evaluation	ADDA scale	12
Risk and prediction of falls	STRATIFY scale	7
Risk of developing pressure ulcers	Braden’s index	6
Quality of sleep	Athens scale	8
Pain assessment	Pain numerical scale	1
Total	-	131

The three large blocks of *Actuasalud* make up 214 items and the mean time used by nurses to conduct the assessment is 35 minutes. Construction of the application’s functionalities had three iterations that served in the development of successive prototypes. For 3 months, a first version (first iteration) was developed, where the proof of concept was carried out, followed by a second iteration with intervention by the scientific team and the nurses who conducted the evaluations; this iteration lasted another three months and testing began on the final version. During this period (third iteration), the first 85 individuals were assessed by nurses from primary care centers and through the meetings of the consensus groups, the result obtained by the platform was evaluated, with respect to the observation by the evaluating nurse regarding the elderly person evaluated. 

### Results of the first assessment performed on elderly individuals with *Actuasalud*

The study included 85 participants who fulfilled the inclusion criteria with a mean age of 74.28 (±6.43) years, the minimum age was 65 years and the most advanced was 91 years. The sociodemographic characteristics revealed prevalence of the female sex (61.2%), no educational level (50.6%), not living alone (55.3%), and 77.6% were retired with income under 900 € (72.9%), and 71.8% did not receive social benefits.

**Table 2:** Sociodemographic variables of the 85 older adults participating in the study

Variables	Categories	*n*	%
Sex	Female	52	61.2
	Male	33	38.8
Educational level	None	43	50.6
	Primary studies	32	37.6
	High school	5	5.9
	University	5	5.9
Living conditions	Lives alone	38	44.7
	Lives with partner	42	49.4
	Lives with children	8	9.4
	Lives with a relative.	3	3.5
	Lives with a caregiver	1	1.2
Usual occupation	Housekeeper	17	20.0
	Employed	0	0.0
	Unemployed	2	2.4
	Retired	66	77.6
Marital status	With a partner	43	50.6
	Without a partner	16	18.8
	Widowed	26	30.6
Monthly income	Without income	7	8.3
	< 900 €	55	64.7
	Between 900 € and 1200 €	10	11.9
	> 1200 €	13	15.5
Social benefits	Yes	24	28.2
	No	61	71.8

Comorbidities showed a series of associated pathologies among which were highlighted arterial hypertension (67.1%; *n* = 57), osteoarthritis and/or arthritis (55.3%; *n* = 47), diabetes (48.2%; *n* =4 1), and falls within the last year (35.3%; *n* = 30). Regarding the analysis of care needs based on frailty, it was found that 44.7% (*n* = 38) of the population was considered Not frail, and 55.3%; *n* = 47 as frail. The cut-off point to differentiate the groups is three or more points according with the frailty scale. The results of the sociodemographic and clinical variables showed statistically significant differences in function of frailty; specifically, a significant association was observed, with lower percentages in non-frail individuals against those who are frail, between being frail and receiving social benefits (25% Vs. 75%; *p =* 0.001), having circulatory problems (18.8% Vs. 81.2%; *p =* 0.041), and taking analgesics regularly (24.2% Vs. 75.8%; *p* = 0.005). 

Regarding the results of the analysis of the battery of instruments to measure frailty-based care needs, those with statistical significance were included; it was noted that frail participants with respect to those not frail had greater care needs in relation to their cognitive state (50% Vs. 50%; *p* = 0.004), basic needs of daily life (75.9% Vs. 24.1%; *p* = 0.012), instrumental activities (45.5% Vs. 54.5%; *p* = 0.002). The quality of life results were broken down into three levels: very good; good, and fair-poor for correlation with frailty and observing very good 26.3% Vs. 73.7%; good 62.9% Vs. 37.1%, and fair-poor 64.5% Vs. 35.5; (*p* = 0.016). Among the responses to the EQ-5D questionnaire, statistical significance was found in the following items, mobility (39% Vs. 61%; *p* = 0.037), hearing (69.4% Vs. 30.6%; *p* = 0.043), falls (42.1% Vs. 57.9%; *p* = 0.048), risk of pressure ulcers (77.8% Vs. 22.2%; *p* = 0.009), and pain (35% Vs. 65; *p*=0.001). 

According with the results of the logistic regression, any status other than retired was considered a risk factor for developing frailty (OR = 4.1, 95% CI = 1.1-15.7); *p* = 0.0355), while not being diabetic (OR = 0.3; 95% CI = 0.1-0.9; *p* = 0.0446) and not having hearing problems (OR = 0.2; 95% CI = 0.1-0.8; *p* = 0.0202) were considered protective factors (Table 3). 

**Table 3:** Multivariate analysis of frailty with the total scores of the questionnaires, and with the sociodemographic variables and comorbidities

Variables	Categories	OR (95% CI OR)	***p-*value**
Age in years	65-74	1	0.0544
	>= 75 years	2.6 (0.9-7.2)	
Sex	Female	1	0.3105
	Male	0.5 (0.2-1.6)	
Usual occupation	Retired	1	0.0355
	Other	4.1 (1.1-15.7)	
Diabetes	Yes	1	0.0446
	No	0.4(0.1-0.9)	
Final hearing score	Requires exploration	1	0.0202
Does not require exploration	0.2 (0.1-0.8)

## Discussion

Evaluating the health status of the elderly population and seeking to identify intervention programs that improve the lives of the elderly is a socio-health need of interest. In this regard, *Actuasalud* has been developed, composed of 14 scales, which assesses 12 dimensions, like cognitive state, frailty, basic and instrumental activities of daily living, feeding, mobility, affective state, social, risk of falls, and pressure ulcers among others to allow autonomous decision making by nurses about the health of the elderly population. Most mobile applications aimed at assessing health problems lack a comprehensive evaluation system of health problems in the elderly, with their use limited to specific purposes.^16^ Nevertheless, mobile applications exist that bear similarities with *Actuasalud*, given that they use similar tools to assess different dimensions with repercussion on frailty and the elderly. A number of applications were identified with a frailty approach, each with different recipients for their use, different clinical criteria, scales and areas, on the one hand, some of these applications are commercialized, which is the case of Dependence Indicators^17^ that include validated scales and permit calculating functional values, with an approach for social work professionals. Other applications have use on development based on comprehensive geriatric assessment, allowing to calculate scale scores and explore clinical recommendations.^18^

For example, there is PowerFrail that mainly evaluates muscle strength^19^ or GeriatriAPP,^18^ which although designed to carry out a comprehensive geriatric assessment, has a clinical approach that includes the prediction of mortality through the Charlson Index, evaluation of risk medications, and a frailty approach focused on the Study of Osteoporotic Fractures, which provides data for non-nursing decision making. According to a Cochrane systematic review published in 2022,^20^ the comprehensive geriatric assessment was designed to elaborate a comprehensive care plan with inconclusive results.

*Actuasalud* was constructed with a preventive disease approach to promote active aging and identify prevention programs relevant to the older population, as recommended in frailty consensus documents, like the updated proposal by the Spanish Ministry of Health for 2022 that includes recent situations, such as the COVID-19 pandemic.^21^ One of the key points in the *Actuasalud* design focuses on the evaluation of needs, early detection of problems, and care continuity, aspects of caring that are the responsibility of nurses and that assessment instruments, such as *Actuasalud*, contribute to decision making by nursing to maintain health in the elderly. According with the digital recommendations by the WHO, digital monitoring of the population is a priority to gather information on health status and its management and electronic health records allow the availability of information and facilitate the search, analysis, and way of sharing information and streamline different phases of care processes. 

Screening for frailty and assessment of human needs in primary care is useful;^18^^,^^22^ however, in actuality in the daily clinical practice screening is not yet carried out homogeneously and, in fact, in Spain, health indicators related with frailty since 2022 seek to gather reliable frailty monitoring information that permits intervening with prevention as of younger ages.^21^ The challenge is to move towards a standardized definition of frailty, as well as the use of electronic problem detection systems that can be easily implemented in clinical practice.^18^^,^^22^ Nevertheless, efforts in implementing tools that support surveillance, awareness, and support systems in decision making are currently the least implemented, behind services such as telephone services and telemedicine. 

The need to use ICTs requires material and human resources that, in turn, increase the cost of care in the short term; nevertheless, in the mid- and long term ICTs diminish care costs.^23^ Not only should we ponder about the search for new tools that include user participation, these must also be cost-effective and in addition to robotics^23^and technological innovation, be able to produce changes in the process of caring for the elderly. Along these lines, the presence of mobile technologies is evident, associated with monitoring daily activities and risk situations,^24^ which are complementary to the contributions made by *Actuasalud*.

Herein, we highlight three points of improvement that can be obtained with the study of frailty: fall prevention, frailty prevention or detection, and improved autonomy to carry out activities of daily life.^8^ Prevention is the key to improve the quality of life of the elderly and sensory-based fall prevention systems are advanced; the majority of fall prevention devices use wireless motion and pressure sensors to determine the risk of falls by comparing data with normal behavioral patterns.^25^ Sensorization of daily activities to report on habits, such as pressure in bed, time away from home, or home activities has also been used.^25^ However, all prevention systems require prior evaluation and screening that starts with the health assessment, so that the results of said assessment permit guiding prevention actions efficiently. This is the principal function by *Actuasalud* that as usability increases, it will allow progress towards automatic care prescription and frailty prediction.

This study concludes that the *Actuasalud* digital platform has been constructed with knowledge from nursing and technology and is accessible to be used by nursing aimed at increasing the use of frailty assessment tools. Its use by clinical nurses contributes in decision making and to support the use and treatment of the assessment data. In the field of nursing education, it can be used in students learning about the use of information technology and computing to evaluate care needs.

With the selection of the dimensions to assess in *Actuasalud*, the dynamics of the interview between professional and user were favored, along with achieving reduced assessment time, improved visualization of patient data, the possibility of the tool to assist the interviewer (through notes in the questions), and, in short, speeding up the interview and data collection process. *Actuasalud* is a preventive tool for use by nurses that allows population screening to detect health problems and frailty states. It consists of a computer system accessible from any mobile device, which permits easily assessing and detecting altered human needs, health and frailty states in population over 65 years of age, with an ordered sequence and with featured functionalities, like management of informed consents, data storage and evaluations, export to databases for subsequent processing, and issuance of graphic reports of the assessments. *Actuasalud* has completed the R+D+I cycle successfully.

This research has a limitation in the recruitment of subjects and their subsequent follow-up, given its difficulty to compete with evaluation systems established in health systems. As future lines of research and development, the implementation of a meta tool is planned to simplify data collection while maintaining the effectiveness of the evaluation that is already being worked on. It is also planned, within the same tool, to advance in the prescription of care and monitoring of individuals assessed to observe possible improvements in their condition or delay in frailty, and even for the system to allow their reference healthcare team to be informed of these warning signs to prevent complications. 

Funding. Conducted within the framework of a business innovation project, funded by Agencia Valenciana de Innovación (AVI) and Universidad de Alicante with a group of experts from the areas of engineering and health. Management Center: Department of Information Technology and Computing. Code:2019/01135/003. Internal reference:AVI1-21.
